# An Overview on Topical Administration of Carotenoids and Coenzyme Q10 Loaded in Lipid Nanoparticles

**DOI:** 10.3390/antiox10071034

**Published:** 2021-06-26

**Authors:** Luciana de Souza Guedes, Renata Miliani Martinez, Nádia A. Bou-Chacra, Maria Valéria Robles Velasco, Catarina Rosado, André Rolim Baby

**Affiliations:** 1 Department of Pharmacy, Faculty of Pharmaceutical Sciences, University of São Paulo, São Paulo 05508-900, Brazil; lsouguedes@hotmail.com (L.d.S.G.); renata.martinez@usp.br (R.M.M.); chacra@usp.br (N.A.B.-C.); mvrobles@usp.br (M.V.R.V.); 2 CBIOS, Universidade Lusófona’s Research Center for Biosciences & Health Technologies, 1749-024 Lisbon, Portugal; catarina.rosado@ulusofona.pt

**Keywords:** carotenoids, coenzyme Q10, lipid nanoparticles, solid lipid nanoparticles, nanostructured lipid carriers, topical administration

## Abstract

Carotenoids and coenzyme Q10 are naturally occurring antioxidant compounds that are also found in human skin. These bioactive compounds have been the focus of considerable research due to their antioxidant, anti-inflammatory, and photoprotective properties. In this review, the current state of the art in the encapsulation of carotenoids and coenzyme Q10 in lipid nanoparticles to improve their bioavailability, chemical stability, and skin absorption is discussed. Additionally, the main findings are highlighted on the cytotoxic and photoprotective effects of these systems in the skin.

## 1. Introduction

It is well-recognized that prolonged exposure to ultraviolet (UV) radiation has harmful effects on human skin, which can cause physiological and biological changes on this tissue, such as DNA damage, premature aging, and cancer, among other effects [1,2,3]. The skin is equipped with an antioxidant network that plays a vital role in its protection from exogenous stressors, e.g., pollution and UV radiation [4]. As the skin is constantly exposed to UV radiation, the endogenous antioxidants present on the outermost stratum corneum layer neutralize reactive oxygen species and free radicals keeping the balance between the formation and neutralization of the reactive species [4,5]. The protective network contains various lipophilic and hydrophilic low molecular-weight antioxidants, like carotenoids (beta-carotene, lycopene, lutein, zeaxanthin, and their isomers), vitamins (A, C, D, and E), enzymes (superoxide dismutase, catalase, and glutathione peroxidase), and other compounds (melanin, flavonoids, lipoic acid, selenium, and coenzyme Q10) [4,6]. These antioxidants act as a protective network and possess a synergistic effect. That is, the antioxidants protect one another from direct degradation during the neutralization process of free radicals and other reactive species [6]. Over time, the antioxidant compounds become depleted and lose their protective effect on the skin. Topical administration can be an alternative to either restore this loss or protect the skin from photodamage [4,6,7,8].

Different types of antioxidant compounds have been topically administered in the skin, such as carotenoids [9,10,11,12,13], coenzyme Q10 [14,15,16,17,18], essential oils [19,20,21], polyphenols [22,23,24,25,26], and vitamins [27,28,29,30,31]. Since only a few active ingredients are effective after topical application due to the barrier function of the skin, lipid nanoparticles have been explored as carriers to enhance bioavailability, skin penetration, and retention of the active ingredients [32,33,34].

Our review aims to provide an outline about the current state of the art of carotenoids and coenzyme Q10 encapsulation in lipid nanoparticles for topical administration. Particularly, the discussion relies on the antioxidant, anti-inflammatory, and photoprotective activities of these bioactives as a strategy to protect skin from UV radiation. In the first section, the two types of lipid nanoparticles, namely solid lipid nanoparticles (SLN) and nanostructured lipid carriers (NLC), are described. Aspects of lipid nanoparticles properties, including stability, loading capacity, and released mechanism, are discussed. The second section discusses the techniques used to produce lipid nanoparticles. Additionally, the advantages and disadvantages of each technique are highlighted. In the third section, the main outcomes of the studies reported in the literature are reviewed and, the last section, summarizes the challenges, future perspectives, and findings on topical administration of carotenoids and coenzyme Q10.

## 2. Solid Lipid Nanoparticles

In the early 1990s, the research groups of Gasco, Müller, and Westesen developed a new type of colloidal system, the solid lipid nanoparticles (SLN), as an alternative to the traditional drug delivery systems, emulsions, liposomes, and polymeric nanoparticles [34,35,36,37,38]. Soon after the SLN discovery, in 1991, this innovative colloidal system was introduced to the scientific community, and the first studies were reported, which immediately attracted attention due to its advantages over other colloidal systems [34]. The number of papers published on this topic increased, and the first review was published in 1995 [39], followed by other important contributions [37,38,40,41,42].

The SLN system consists of spherical particles in the nanometer range, 50–1000 nm, whose shells are made up of solid lipid or a mixture of solid lipids that are dispersed in an aqueous surfactant solution [34,36,38,43,44]. The SLN are prepared from highly purified triglycerides, complex glyceride mixtures, fatty acids, steroids and waxes, which are solid at body and room temperature [34,36,40,44]. The solid lipid content of the colloidal system ranges from 0.1–30.0% (*w*/*w*) and it is stabilized in aqueous media by 0.5–5.0% (*w*/*w*) of surfactants [42,45,46,47]. A wide variety of surfactants are used in the production of SLN, such as bile salts, ethoxylated alcohols, fatty acids, phospholipids, poloxamers, polyethylene glycols, polysorbates, polyvinyl alcohols, quaternary ammonium compounds, sorbitan esters, and tyloxapol [35,45,47,48,49].

Lipophilic and hydrophilic active ingredients can be entrapped in the SLN [45]. This delivery system offers some advantages, such as protection of labile compounds against degradation, high biocompatibility, and controlled release of the active ingredient [36,40,42,43,45]. Furthermore, when applied to the skin, the SLN increase the active ingredient bioavailability due to the small size of the lipid nanoparticles that forms a thin film over the skin, ensuring a closer contact with the stratum corneum, which, consequently, enhances the amount of the active ingredient that could penetrate the skin. In addition, the film formed can develop an occlusive effect that could reduce water loss. Studies demonstrated that the occlusion increases with a decrease in the lipid melting point and the particle size and an increase in the particle crystallinity [21,42,43]. Another SLN interesting property is its sun protective effect, i.e., SLN could act as a physical UV filter, which would offer an additional benefit for topical applications [42,44,47].

The structure and morphology of the nanoparticles are affected by the system constituents. The type of lipids, their physicochemical and self-emulsifying properties, and crystallization velocities affect the shape, size, and stability of the SLN. For instance, nanoparticles prepared with highly pure lipids, such as tristearin or cetyl palmitate, have a more cubic shape since the lipid structure reorganizes in a way that is similar to a brick wall. On the other hand, the use of crude mixtures leads to nanoparticles with a spherical shape due to the different chain lengths of the lipid molecules [40,50,51]. In addition, the choice of the constitutive lipids impacts the loading capacity and the release profile of the active(s) and the skin penetration/permeation. In a similar manner, the surfactant type and concentration play an important role in SLN formation. High surfactant concentration reduces the surface tension and facilitates the particle partition during the homogenization process. Furthermore, SLN dispersions containing surfactant mixtures have smaller sizes and higher storage stability compared to samples with only one surfactant [40,50]. Besides the lipids and surfactants, SLN formation is influenced by the solubility and concentration of the active ingredient in the lipid matrix and by the production method [47].

The incorporation of an active ingredient in SLN depends on its chemical nature, the constituents of the lipid matrix, and the production parameters. Three incorporation models have been proposed in the literature based on the location and distribution of the active ingredient within the lipid core [51,52]. In the SLN type I or homogenous matrix model, the active ingredient is molecularly dispersed in the lipid core or is present as amorphous clusters. This SLN type allows for a sustained release of the active ingredient. The SLN type II or drug-enriched shell model is obtained when the active ingredient solubility in the lipid phase is lower than in the water phase. Owing to this limited solubility, a drug-free or drug-reduced lipid core is formed as the nanoemulsion is cooled down to room temperature leading to the formation of a drug-enriched shell. For this SLN model, as the active ingredient is mainly located on the nanoparticle surface, the short distance of diffusion results in a fast-initial release. The SLN type III or drug-enriched core is formed when the concentration of the active ingredient is close to or at its saturation solubility in the melted lipid. During cooling, the active ingredient precipitates before the lipid recrystallizes, which leads to supersaturation of the active ingredient in the melted lipid—further cooling results in the formation of a lipid shell covering the active ingredient. A fast-initial release followed by a more gradual release is observed for this SLN type, whose mechanism is controlled by the mobility of the active ingredient in the solid lipid matrix [51,52,53,54,55,56].

Besides the location of the active ingredient in the SLN matrix, the release mechanism is dependent on other parameters such as the polymorphic state of the lipid matrix, the type and concentration of the surfactant, and the production conditions [45,52,54]. Polymorphism is the ability of a molecule to crystallize in more than one crystalline species that possess different internal lattice structures [51]. The internal structure of the lipid matrix mainly occurs in three specific types of sub-shells, the polymorphs α, β’, and β. The α-form is metastable and possesses hexagonal sub-shell packing in which the fatty acid chains are perpendicular to the methyl group plane. The β’-form has intermediate stability and possesses orthorhombic sub-shell packing where the fatty acid chains are tilted with respect to the methyl group plane, while the β-form has greater stability and possesses triclinic parallel packing [45,51,57]. In the nanoparticles, the lipid recrystallizes at least partially in the α-form and undergoes a polymorphic transition to more stable forms during storage [45,51]. This phenomenon is dependent on the degree of homogeneity of the triglycerides. For instance, short-chain saturated monoacid triglycerides undergo more quickly from the α-form to the β-form than triglycerides with long chains [45,55].

As mentioned previously, the release mechanism is also dependent on the surfactant type and concentration. The interaction between the surfactant and the outer shell of the nanoparticles affects the SLN structure and its concentration the extent of the initial burst release, e.g., low surfactant concentration leads to minimal burst while higher surfactant concentration increases the burst effect. The initial burst can be modulated by the production conditions, a minimal burst can be obtained by employing low temperature, which minimizes the partition of the active ingredient between the lipid and aqueous phases and results in a homogenous distribution of the active ingredient in the lipid matrix before homogenization. As the nanoparticles are formed, this homogeneous distribution is retained and, therefore, reduces the burst effect [38,52,54]. 

Even though SLN has demonstrated several advantages as a colloidal carrier, this delivery system has some limitations. For instance, a low loading capacity due to a densely packed crystal network that allows little room for molecule accommodation, active ingredient expulsion during storage as a consequence of the polymorphic transformation to a more stable crystal form, presence of alternative colloidal structures (micelles, liposomes, mixed micelles, and drug nanocrystals), and high-water content (70–99.9%), which requires its incorporation into semisolid formulations, like hydrogels or creams. In addition to those, SLN dispersion may undergo a gelation phenomenon where its viscosity increases during the cooling process resulting in a viscous gel, which consequently leads to an increase in particle size and particle agglomeration. Owing to these limitations, the second generation of lipid nanoparticles was developed to resolve these challenges [40,52,56,58,59].

## 3. Nanostructured Lipid Carriers

In 1999, the second generation of lipid nanoparticles, the nanostructured lipid carriers (NLC), were developed to overcome some of the drawbacks encountered in SLN [34,36,41]. NLC differs from the first generation of lipid nanoparticles by the solid matrix composition, which comprises a mixture of solid and liquid (oil) lipids that are solid at room temperature. Structurally, the particle size of this colloidal system ranges from 10 to 1000 nm [36,42,46,47]. The constituents of the lipid matrix are biocompatible and biodegradable lipids that exhibit excellent tolerability and are blended in a ratio ranging from 70:30 up to 99.9:0.1 of solid lipid to oil [36,42,46,47]. The solid lipids that comprise the NLC matrix are the same used in SLN, while the oils may be fatty alcohols, medium-chain triglycerides, paraffin oil, and squalene. Additionally, fatty acids, such as oleic, linoleic, and decanoic acid may be employed due to their properties as penetration enhancers. Hydrophilic, lipophilic, and amphiphilic surfactants are used to stabilize the NLC dispersions, such as poloxamers, sorbitan esters, and phosphatidylcholines that may be combined to prevent particle aggregation [60].

In contrast to the relatively perfect crystal lattice of the SLN system, the addition of oil in the lipid phase of the NLC results in imperfections in the solid matrix, i.e., the oil distorts the formation of perfect lipid crystals, which increases the loading capacity of the lipid nanoparticles and minimizes active ingredient expulsion during storage [41]. Another advantage of the NLC is that this colloidal system allows highly concentrated dispersions of lipid nanoparticles (>30–95%). These advantages make NLC a biocompatible carrier for several types of active ingredients intended for pharmaceutical and cosmetic applications [36,50,56,61].

Similar to SLN, incorporation of the active ingredient in the NLC is influenced by its solubility in the melted lipids and the physicochemical properties of the solid matrix. The active ingredient distribution in the lipid matrix can be described by three models that differ by the type of lipids used in NLC production [51,56]. The NLC type I or the imperfect crystal model is formed by mixing spatially different lipids, e.g., the mixture of mono-, di-, triacylglycerols that create imperfections in the crystal order due to the different chain lengths of the fatty acids. The imperfections and voids in the matrix are able to accommodate the active ingredient in molecular form and amorphous clusters, which increases the active payload. The NLC type I is obtained when solid lipids are mixed with small amounts of oils, which results in a less order matrix with more available spaces to accommodate the active ingredient. The NLC type II or the amorphous model is obtained when oils that do not recrystallize after homogenization or cooling are employed, such as medium-chain triglycerides, isopropyl myristate, hydroxy octacosanyl hydroxy stearate, and dibutyl adipate. By using these oils, recrystallization to the beta-form is prevented by maintaining the solid lipid matrix in an amorphous state, which consequently minimizes expulsion of the active ingredient during storage. The NLC type III or multiple models are characterized by the presence of small oil nanocompartments in the solid lipid matrix. This NLC structure is formed by mixing solid lipids with oils in a ratio that exceeds the oil solubility resulting in the formation of tiny oil droplets in the solid lipid matrix. Medium and long-chain triacylglycerols and oleic acid are used as the liquid lipids for the production of this type of NLC, which is advantageous for incorporation of active ingredient that exhibits higher solubility in oils than in solid lipids [21,37,51,52,55,56].

**Figure 1:**
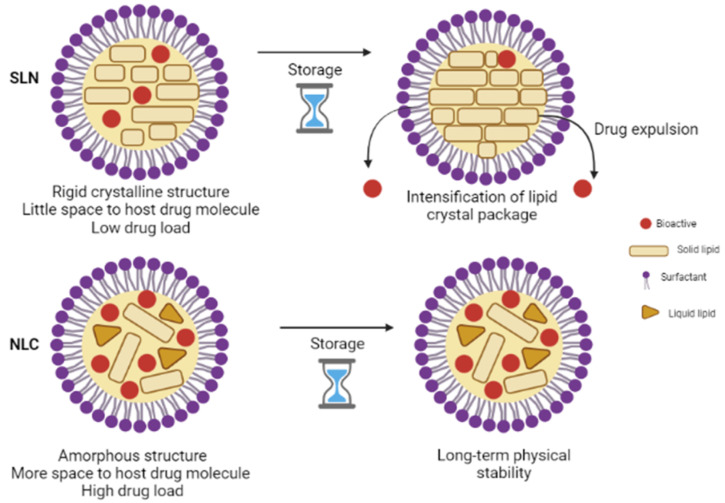
Comparative structure of SLN and NLC.

Analogous to SLN, the release mechanism from NLC is governed by several parameters. In addition to those mentioned in the SLN section, the release of the active ingredient is also dependent on the liquid lipid content as its addition reduces the crystallization process of the lipid matrix providing much more flexibility to achieve a desired prolonged-release [56,60,62]. NLC imparts many advantages over SLN (Figure 1). For instance, the potential issues associated with SLN, such as limited loading capacity and expulsion of the active ingredient during storage, are avoided or minimized by the NLC system [63]. Furthermore, ease of production and feasibility of scale-up has led to the fast development of this colloidal system [64,65].

## 4. Production Techniques 

Several techniques have been reported in the specialized literature that can be used in the production of both SLN and NLC, such as high-pressure homogenization (HPH) [37,38,40,45,53,66], *o*/*w* microemulsion [67,68,69], microemulsion cooling [67,68,69,70], solvent emulsification/evaporation [34,35,40,71,72], emulsification/diffusion [34,35,45,71,72], solvent injection or solvent displacement [45,52,54,56,73], membrane contactor [45,55,74,75], phase-inversion temperature [51,76,77,78], double emulsion (*w*/*o*/*w*) [45,56], coacervation [53,73,79,80], spray-drying [53,81], electrospray [53,81], and supercritical fluid [53,73,81,82]. Among these techniques, the most used are the HPH developed by Müller and Lucks [37] and the microemulsion introduced by Gasco [34,38,40,45].

In the HPH method, the active ingredient is dispersed into the lipids melted at 5–10 °C above their melting temperatures and then mixed with an aqueous surfactant solution at the same temperature. This pre-emulsion is prepared in a high shear mixing device (Ultra Turrax, for example). Then, the obtained pre-emulsion is homogenized in a piston-gap homogenizer; the pre-emulsion is pushed under high pressure (100–2000 bar) through a narrow gap (in the range of few microns) for 3–5 times. The high-shear stress and the cavitation forces break down the particles to the submicron range. The particle sizes are influenced by the operating parameters, pressure and number of cycles; an increase on these parameters result in an increase in the particle sizes. The homogenization process can be performed at high or low temperatures, and it is called hot-HPH and cold-HPH, respectively [37,38,40,45,66].

Hot-HPH is carried out at temperatures above the melting points of the lipids, which results in small particle sizes due to a decrease in the pre-emulsion viscosity. Lipid nanoparticles are formed after cooling the sample to room temperature or below room temperature. Cold-HPH is usually used for thermo-labile active ingredients and also for hydrophilic active ingredients that would partition into the lipophilic and hydrophilic phases during the hot-HPH method. The first step in the cold-HPH method is similar to the hot-HPH; the active ingredient is dispersed in the melted lipids followed by a fast cooling that can employ dry ice or liquid nitrogen. Next, the solid is milled in a ball or mortar milling to obtain particles in the 50–100 micron range. The powder is dissolved in a chilled aqueous surfactant solution, and the obtained pre-emulsion is submitted to the HPH method at or below room temperature. In the homogenizer, the microparticles are broken down into nanoparticles. To avoid melting of the lipids in the equipment, a large temperature difference between the melting points of the lipids and the homogenizer temperature is required, since during the homogenization process, the temperature increases 10–20 °C per cycle. Large particle sizes and broad size distributions are obtained when cold-HPH is employed [37,38,40,45].

Another method employed in the production of lipid nanoparticles is the one developed by Gasco [83], the *o*/*w* microemulsion method. In this method, the active ingredient is dissolved in the melted lipids; the surfactant and co-surfactant are dispersed in water heated at the same temperature as the lipid phase. The two phases are mixed, and the obtained microemulsion is dispersed in cold water at 2–10 °C under mild stirring to ensure that the resulting particle sizes are due to precipitation and not mechanically induced by the stirring process. In these conditions, the microemulsion is broken, and the lipid nanoparticles precipitate. The volume ratio of the hot microemulsion to cold water may vary from 1:25 to 1:50. In contrast to the HPH technique, a low lipid content is obtained by the microemulsion procedure due to the dilution step in cold water. This method is inexpensive, fast, and no special equipment is required. However, the dilution step could hamper a potential industrial use since an excessive amount of water should be removed from the final product [34,38,40,45,84,85]. A variant of the microemulsion method, the microemulsion cooling, has been reported by Koziara et al. [67]. In this method, constituents of the lipid and aqueous phases are added to the same vessel and heated at mild temperatures. The nanoparticles are obtained by cooling the undiluted *o*/*w* microemulsion to room temperature while stirring. Moderate operating temperature and easy manufacturing process are the main advantages of this method [67,68,69,70].

Other important techniques are based on the use of organic solvents, and the production of lipid nanoparticles is carried out at mild operating temperatures, which are particularly useful for thermosensitive compounds [34,53]. In the solvent emulsification/evaporation method, the active ingredient and the lipids are dissolved in a water-immiscible solvent, e.g., chloroform, dichloromethane, or cyclohexane, emulsified in an aqueous solution, followed by solvent evaporation under reduced pressure, which results in precipitation of the lipid phase in the aqueous medium. In this method, the concentration of the lipids in the organic phase affects the particle size, and an increase in their concentration may influence the homogenization efficiency due to an increase in the organic phase viscosity. An advantage of this method is the avoidance of thermal stress, while a disadvantage is the toxicological issues that may arise from solvent residues [34,35,40,71,86].

In the solvent emulsification/diffusion or solvent displacement method, a water-miscible solvent, acetone, dimethyl sulfoxide, or ethanol, is added to the molten lipids. Then, the organic phase is dispersed in an aqueous surfactant solution kept at the same temperature. The organic phase diffuses to the water phase, and subsequent cooling results in nanoparticle precipitation [34,35,45,71,72]. Alternatively, the molten lipids can be injected through an injection needle into the stirring aqueous phase. Then, the resulting dispersion is filtered through a paper filter to remove any lipid excess [45,52,54,56,73,87].

The double emulsion (*w*/*o*/*w*) method is mainly used for the production of lipid nanoparticles loaded with hydrophilic active ingredients. First, a *w*/*o* emulsion is prepared to employ the solvent emulsification/diffusion method without the cooling step. Then, the *w*/*o* emulsion is dispersed in a surfactant solution at 2–3 °C under stirring, which results in the diffusion of the organic solvent to the aqueous phase and the nanoparticle precipitation [45,54,56,85,88]. 

Other alternative approaches have been reported in the literature [55,75,76,78]. In the membrane contactor method, the lipid phase is heated at a temperature above the melting point of the lipids and then pressed through membranes. The small droplets formed are swept away from the membrane pore outlets by a continuous aqueous phase that circulates inside the membrane module. The nanoparticles are formed by cooling the dispersion to room temperature. The operating parameters, the temperature of the lipid and the aqueous phases, pressure, size of the membrane pores, and cross-flow velocity of the circulating water affect the flux of the lipid phase and the size of the nanoparticles. An increase in the lipid phase flux is obtained with a decrease in lipid content, an increase in temperature (above the melting point of the lipids), an increase in pressure and use of membranes with large pore size. Regarding the size of the nanoparticles, it can be decreased by reducing the lipid content, increasing the temperature of the lipid phase, decreasing the temperature of the water phase, and increasing the cross-flow velocity. The feasible production and scaling-up are some advantages of this method [45,55,74,75].

The phase-inversion temperature (PIT) method was proposed to minimize the use of organic solvents and surfactants. This method involves two steps, in the first step, all components, lipophilic and hydrophilic constituents, salt and water, are mixed together and heated above the melting point of the lipids. Then, progressive cooling is carried out 3 times to determine the cooling-dilution temperature. This temperature indicates the beginning of the phase-inversion zone in which an inversion from an *o*/*w* to a *w*/*o* emulsion occurs. The thermal range of the phase-inversion zone varies with the salinity of the medium and is adjusted according to the formulation. In the second step, the emulsion is diluted with cold water to break the system, and the fast cooling process leads to nanoparticle formation [51,77]. A variant of the PIT method has been reported in other studies [76,78]. In those studies, the lipid and aqueous phases are heated separately, and the excess of surfactants is removed by centrifugation.

In the high-shear homogenization and/or ultrasonication method, lipids are heated above their melting point and dispersed in a surfactant solution at the same temperature under high-speed stirring. Then, the emulsion is ultrasonicated to reduce the droplet sizes and allowed to cool to room temperature. Concentrated nanoparticle dispersion can be obtained by ultracentrifugation. Low dispersion quality, presence of microparticles, and metal contamination are some of the disadvantages of this method [40,52,56,89,90,91].

Another method for the production of lipid nanoparticles is the coacervation method patented by Battaglia et al. [80], which is based on a slow interaction between a fatty acid salt and an acid solution (coacervating solution) in the presence of a polymeric non-ionic surfactant. First, the surfactant solution is prepared by heating the polymer in water followed by its cooling to room temperature. Then, the fatty acid salt is dispersed in the surfactant solution and heated under stirring just above its Krafft point, that is, above the temperature in which the last crystal of the fatty acid salt dissolves and the solution becomes clear. An acidifying solution is added drop wisely up to pH 4.0, which results in nanoparticle precipitation. In the subsequent step, the dispersion is cooled in a water bath under stirring. Sodium salts of fatty acids, such as, sodium stearate, sodium palmitate, sodium myristate, sodium arachidate, and sodium behenate, are used with concentration that varies from 1 to 5% *w/w*. Poly vynil acetate/poly vynil alcohol and polyoxyethilene/polyoxypropylene copolymers, dextrans, hydroxypropylmethylcellulose, and non-ionic gums are normally selected to compose these systems. This method allows the incorporation of the active ingredient without using complex equipment and harmful solvents. Additionally, particle size can be modulated by the lipid concentration and the surfactant type [53,73,79,92].

In the microwave-assisted microemulsion technique, the constituents of the lipid and aqueous phases are mixed in a single vessel, kept under stirring, and subjected to controlled microwave heating at a temperature above the melting point of the lipids. The microemulsion is dispersed in cold water, 2–4 °C, to generate the nanoparticles [93,94,95].

Techniques to produce lipid nanoparticles in a solid powdered state have been reported in the literature. An extensive review on this topic was published by Berton et al. [81], which included spray-drying, electrospray, and supercritical fluid techniques. In the spray-drying method, a liquid feed is converted into a dried particulate form by a one-step process. The liquid feed is comprised of an organic solvent solution that is atomized to a spray form and subsequently put into thermal contact with hot gas to remove the solvent. Following the solvent removal, the formed nanoparticles are separated from the gas by a cyclone, an electrostatic precipitator, or a bag filter. Small and homogenous particle sizes are obtained by this method, which is particularly useful for thermo-labile active ingredients and compounds with a low melting point or glass transition temperature [53,81]. In the electrospray method, lipids are dissolved in an organic solvent and introduced in a syringe with a metal capillary connected to a high-voltage power supply as one electrode and a metal foil collector at the opposite side as a counter electrode. The lipid solution travels through the applied electric field, emerges from the nozzle, and forms droplets due to the surface tension. By selecting suitable conditions, voltage, and liquid flow rate, droplets with a narrow size distribution can be generated [53,81,96].

Different methods employing supercritical fluid have been developed for nanoparticle production [53,81,82]. In the supercritical fluid extraction of emulsion (SFEE) method, an *w*/*o* emulsion is prepared by solubilizing the lipid phase in an organic solvent (for example, chloroform) with the addition of a surfactant. In a subsequent step, the lipid phase is dispersed in an aqueous surfactant solution, which is passed through a high-pressure homogenizer to form an o/w emulsion. Then, the o/w emulsion is introduced in an extraction column from the top, and a supercritical fluid, usually CO_2_, is introduced from the bottom. In contact with a supercritical fluid, the organic solvent is extracted from the o/w emulsion resulting in nanoparticle precipitation. This method enables effective removal of the solvent and produces nanoparticles with a uniform size distribution [52,53,73,82]. In the gas-assisted melting atomization (GAMA) method, lipids are introduced in a mixing chamber and kept in contact with a supercritical fluid. The mixture is forced through a nozzle, and the fast decompression drives the solution to a supersaturated state resulting in nanoparticle precipitation. In a subsequent step, the nanoparticles are collected and dispersed in water [53,97,98]. Similar to the GAMA method, the particle from gas saturated solution (PGSS), the rapid expansion of supercritical solution (RESS), the aerosol solvent extraction system (ASES), and the solution enhanced dispersion by supercritical fluid (SEDS) methods employ decompression through a nozzle to produce the lipid nanoparticles [52,81].

## 5. Carotenoid Encapsulation in Lipid Nanoparticles

Carotenoids are natural pigments synthesized by plants, animals, and microorganisms that are widely spread in nature. There are more than 600 carotenoids with structural variants, which are divided into two groups, namely carotenes and xanthophylls. Carotenes are oxygen-free molecules whose structure contains only a hydrocarbon chain without any functional group with a general formula C_40_H_56_. Carotenes occur in several isomeric forms, such as alpha, beta, gamma, delta, epsilon, and zeta forms. Xanthophylls are the oxidized derivatives of carotenes with a general formula C_40_H_56_O_2_. The oxygen group in the xanthophyll structures can be found as alcohol, ketone, or alcohol esters. Owing to these groups, xanthophylls are more polar than carotenes [5,48,99,100,101].

Carotenoids can also be classified as pro-vitamin A compounds, that is, those that can be converted into vitamin A in the human body, such as alpha-carotene, beta-carotene, and beta-cryptoxanthin, and non-pro-vitamin A compounds like lutein and lycopene. Carotenoids are not produced by the human body and, therefore, must be consumed in the diet [5,48,99]. Approximately 40 carotenoids are present in a typical human diet, and about 20 carotenoids have been identified in human blood and tissues. Among the 20 carotenoids found in the human body, almost 90% is represented by beta-carotene, alpha-carotene, lycopene, lutein, and cryptoxanthin [101,102]. A diet rich in carotenoids has been related to a lower incidence of cancer, cardiovascular diseases, and diabetes [101,103]. 

Carotenoid ability to reduce the risk of several ailments and age-related biological transformations has been attributed to its antioxidant and anti-inflammatory properties [104]. In the skin, they act as a protective barrier to UV radiation and accumulate mostly in the epidermis, whose amount depends on dietary intake and supplementation [105,106,107,108]. Likewise, topical administration of carotenoids to the skin and their role in photoprotection has been investigated [106,109]. However, the effective use of these lipophilic compounds is limited by their low solubility in water and degradation upon exposure to heat and oxygen. To overcome these limitations, carotenoid encapsulation in lipid nanoparticles has been used as a successful strategy to enhance their solubility and protect them from degradation [48,110]. Table 1, Table 2, Table 3 and Table 4 present studies reported in the scientific literature on carotenoid encapsulation in SLN and NLC for topical administration. The main experiments and outcomes on the physicochemical characterization of these colloidal carriers are highlighted. Additionally, in vitro studies on carotenoid release mechanism and in vitro and in vivo evaluation of antioxidant and cytotoxic effects of the investigated systems are discussed.

For most of the studies, high percentages for the entrapment efficiency were obtained. Investigation on the short and long-term stability showed that the colloidal systems were more stable at room temperature or below. Moreover, the formulations showed good photostability. The studies demonstrated that SLN and NLC are good vehicles to protect carotenoids from degradation. Other important findings were the effect of the system constituents, solid, and liquid lipids and surfactants, on the physicochemical properties of the nanoparticles. The in vitro release studies revealed that the release mechanism was characterized by an initial burst followed by a prolonged release. Carotenoid antioxidant properties were enhanced upon encapsulation, while the cytotoxic studies suggested that the investigated systems were well tolerated. Furthermore, SLN and NLC improved carotenoid penetration into the upper layers of the skin. 

## 6. Coenzyme Q10 Encapsulation in Lipid Nanoparticles

Coenzyme Q10 (CoQ10), also known as ubiquinone or ubidecarenone, is a liposoluble vitamin-like compound that plays a relevant role in several biochemical processes, such as the production of cellular energy in the form of adenosine triphosphate, the mitochondrial electron transport chain, and the inhibition of cell membrane peroxidation [23,124,125,126,127]. CoQ10 is part of a homologous series of substances that share a common benzoquinone ring structure, differing in the length of the isoprenoid side chain. The Q in CoQ10 name refers to the quinone group and, the number, the isoprene units in the side chain; each isoprene unit contains 5 carbons [126,127,128,129,130]. In humans and a few other mammalian species, the side chain contains 10 isoprene units [129,131]. 

CoQ10 is the only lipid-soluble antioxidant produced within the body. Its level in human tissues is higher in organs with high metabolism rates like the heart, kidney, and liver, where it functions as an energy transfer molecule. In the skin, the CoQ10 level is 10-fold higher in the epidermis than in the dermis. CoQ10 has demonstrated positive effects in the treatment of cardiovascular, neuromuscular, and infertility diseases. Regarding its cosmetic application, CoQ10 has shown the ability to reduce photoaging and wrinkle depth, whose effects could be related to its ability to increase the production of basal membrane components, fibroblast proliferation and to protect the cell against oxidative damage [15,132,133]. However, its level decreases with age and after exposure to UV radiation [134,135,136].

CoQ10 exists in two redox forms, the oxidized and reduced forms. The oxidized form, ubiquinone, is synthesized within cells and is a component in the mitochondrial electron transport chain, while the reduced form, ubiquinol, acts as a potent antioxidant outside the mitochondrial membrane, which can inhibit both the initiation and propagation steps of lipid peroxidation and regenerate other antioxidants like vitamin C and E [126,136,137,138]. 

CoQ10 is almost insoluble in water, chemically instable, and easy to degrade when exposed to light [124,138]. Encapsulation of CoQ10 in lipid nanoparticles is one of the approaches used to improve its bioavailability and enhance its chemical and physical stability [14,16]. Table 5, Table 6 and Table 7 report the studies found in the literature on encapsulation of CoQ10 in SLN and NLC for topical application. Regarding the physicochemical characterization, it was demonstrated that high CoQ10 concentration prevented nanoparticle formation. The long-term stability studies (1 year) indicated a slight change in particle size, zeta potential, and polydispersity index, whereas a significant decrease in CoQ10 loading was observed. It is interesting to highlight that the structural characterization of the CoQ10-SLN system showed that a large portion of CoQ10 integrated homogenously with the solid lipid matrix.

The in vitro permeation studies showed an increase in CoQ10 concentration in the epidermis. Additionally, the results indicated that the amount of CoQ10 in the skin was affected by the lipid matrix composition and the occlusive properties of the nanoparticles. The studies showed that CoQ10 loaded in lipid nanoparticles exhibited antioxidant and photoprotective activities. Moreover, evaluation of skin hydration and viscoelasticity indicated an increase in these skin features after administration of CoQ10 loaded in lipid nanoparticles. The ability of CoQ10 to improve skin hydration and elasticity have been explored in several commercial products containing CoQ10-NLC [60,61,139].

**Table 5:** An overview of CoQ10 encapsulation in solid lipid nanoparticles (SLN).

Ingredients and Method	Parameters	Outcomes	Reference
1. CoQ10 2–90% 2. *Lipid phase:* triglyceride, Lipoid S 100, sodium glycocholate or triglyceride, Lipoid S100, tyloxapol 3. *Aqueous phase:* thiomersal, glycerol, and water 4. High-pressure homogenization	1. Physicochemical characterization	1. CoQ10 concentration affected the melting and crystallization behavior of the lipid. Higher CoQ10 concentration prevented nanoparticle formation. Slow-release was obtained at a low CoQ10 concentration	[16]
1. CoQ10 0.01% (*w/w*) 2. *Lipid phase:* beeswax, cetyl alcohol, cetyl palmitate, Compritol 888 ATO, stearic acid, stearyl alcohol, or spermaceti 3. *Aqueous phase:* surfactants (Tego Care 450, Tween 20 or Tween 80) and water 4. High-pressure homogenization	1. Stability (6 months) 2. In vitro release (continuous flow diffusion cells, cellulose nitrate membrane) 3. In vitro skin hydration and viscoelasticity (25 females)	1. Stability was affected by lipid and surfactant combination 2. Fast release was observed at the initial stage followed by a prolonged release 3. An increase in skin humidity and elasticity was observed	[134]
1. CoQ10 5 mg 2. *Lipid phase:* Compritol 888 ATO 3. *Aqueous phase:* Poloxamer 188, Tween 80 and water 4. High-shear homogenization	1. Entrapment efficiency 2. Cytotoxicity (MTT assay, human dermal fibroblasts) 3. Intracellular reactive oxygen species (ROS) accumulation	1. Entrapment efficiency was > 89% 2. CoQ10-SLN showed biocompatibility towards fibroblasts up to 50 μM 3. CoQ10-SLN showed no protective effect against ROS accumulation	[140]
1. CoQ10 0.02% (m/V) 2. *Lipid phase:* Compritol 888 ATO 3. *Aqueous phase:* Poloxamer 188, Tween 80 and water 4. High-shear homogenization	1. Entrapment efficiency 2. Production yield 3. Antioxidant activity (TEAC method) 4. Rheological studies 5. Ex vivo diffusion (Wistar-albino rat)	1. Entrapment efficiency was 89% 2. Production yield was 94% 3. SLN protected CoQ10 from degradation 4. No changes in the gel rheological features was observed after SLN incorporation 5. CoQ10 penetration into the skin increased	[141]
1. CoQ10 5, 10 and 50% (*w/w*) 2. *Lipid phase:* Dynasan 114 and soy bean lecithin Lipoid S100 3. *Aqueous phase:* sodium glycocholate and water 4. High-pressure homogenization	1. Influence of the SLN crystalline state on loading capacity and mobility of CoQ10	1. SLN was stable at all concentrations. Mobility of CoQ10 molecules was observed	[142]
1. CoQ10 4.8% 2. *Lipid phase:* cetyl palmitate 3. *Aqueous phase:* Tego Care 450 and water 4. High-pressure homogenization	1. Internal structure investigation (Solid-state NMR)	1. Large portion of CoQ10 occurred in a homogenous mixture with solid lipid	[143]

**Table 6:** Studies on CoQ10 encapsulation in nanostructured lipid carriers (NLC) employing high-pressure homogenization.

Formulation Ingredients	Parameters	Outcomes	Reference
1. CoQ10 0.07% (*w*/*w*) 2. *Lipid phase:* cetyl palmitate and coconut oil 3. *Aqueous phase:* Tween 80 and water	1. Entrapment efficiency 2. Long-term stability (1 year) 3. In vitro release (dialysis bag) 4. Rheological study 5. Antioxidant activity (DPPH assay) 6. Ex vivo skin permeation (tape stripping, female Wistar rats) 7. Anti-inflammatory activity (Carrageenan-induced rat paw edema model)	1. Entrapment efficiency > 96% 2. No significant change was observed in particle size, zeta potential, and polydispersity index 3. Fast release observed at the initial stage followed by a prolonged release 4. CoQ10-NLC viscosity was greater than w/o cream 5. Antioxidant activity of NLC loaded with high CoQ10 content was higher than free CoQ10 6. CoQ10-NLC exhibited lower penetration than w/o cream 7. CoQ10-NLC showed the most potent anti-inflammatory effect	[14]
1. CoQ10 1% (*w*/*w*) 2. *Lipid phase:* glycerin monostearate and caprylic capric triglyceride 3. *Aqueous phase:* Cremophor A25, dipolyhydroxystearate (P135), and water	1. Large-scale production (25 kg/h) 2. Particle size 3. Stability (6 months)	1. Production line enabled particle size below 210 nm 2. Particle size was influenced by pre-emulsification temperature, homogenization pressure, and homogenization cycles 3. All batches at room temperature and below were stable	[17]
1. CoQ10 2–5% 2. *Lipid phase:* glycerin monostearate, glyceride, and ethanol 3. *Aqueous phase:* Span 20 and water	1. CoQ10-NLC photo-stability (5 months) 2. Nanoparticle morphology 3. Cell cytotoxicity (MTT assay, HeLa cells)	1. CoQ10-NLC degradation was lower than free CoQ10 2. CoQ10-NLC exhibited non-spherical shaped nanoparticles 3. Cell viability decreased with an increase in CoQ10-NLC concentration	[127]
1. CoQ10 2.4 and 4.8% (*w/w*) 2. *Lipid phase:* cetyl palmitate and Miglyol 812 3. *Aqueous phase:* Tego Care 450 and water	1. Rheological studies 2. Accelerated (exposure to day light, 28 days) and long-term (1 year) stability 3. In vitro skin permeation (Franz diffusion cells, human epidermis)	1. Spatial arrangement of lipid molecules was observed after NLC incorporation into hydrogels 2. Accelerated stability showed a significant decreased in CoQ10 content while long-term stability had a slightly decrease 3. Amount of CoQ10 in the skin was affected by the oil content and the occlusive effect	[144]
1. CoQ10 5% (*w*/*w*) 2. *Lipid phase (NLC):* cetyl palmitate and Miglyol 812 3. *Aqueous phase (NLC):* Tego Care 450 and water 4. *Lipid phase (Ultra-small NLC):* cetyl palmitate and Miglyol 812 5. *Aqueous phase (Ultra-small NLC):* Tween 80, Span 20, and water	1. In vitro release (Franz diffusion cells, cellulose acetate membrane) 2. In vitro antioxidant activity (DPPH assay) 3. In vivo antioxidant activity (Kirial test)	1. Ultra-small NLC showed higher release 2. Higher antioxidant activity was observed for ultra-small NLC in both in vitro and In vivo studies	[145]
1. CoQ10 5% 2. *Lipid phase (NLC):* cetyl palmitate and Miglyol 812 3. *Aqueous phase (NLC):* Tego Care 450 and water 4. *Lipid phase (Ultra-small NLC):* cetyl palmitate and Cetiol OE 5. *Aqueous phase (Ultra-small NLC):* Span 20, Tween 80 and water	1. Cellular uptake (human keratinocytes HaCaT cells) 2. Cell viability assay (XTT assay, human keratinocytes HaCaT cells) 3. Radical formation after irradiation with UVA/UVB light	1. Distribution of NLC within the cytoplasm was observed 2. Low CoQ10 content showed to be non-toxic 3. Strong reduction on radical formation was observed with ultra-small NLC	[146]
1. CoQ10 4.5% (*w*/*w*) 2. *Lipid phase:* cetyl palmitate, Miglyol 812, and Tego Care 450 3. *Aqueous phase:* water	1. Selection of preservatives	1. Physical stability of the NLC dispersions was affected at different levels by the type of the preservatives	[147]
1. CoQ10 5% (*w*/*w*) 2. *Lipid phase (NLC):* cetyl palmitate and Miglyol 812 3. *Aqueous phase (NLC):* Tego Care 450 and water 4. *Lipid phase (Ultra-small NLC):* cetyl palmitate, cetiol OE and Span 20 5. *Aqueous phase (Ultra-small NLC):* Tween 80 and water	1. In vitro skin diffusion (Franz diffusion cells, porcine skin) 2. In vitro skin permeation (tape stripping, porcine ear skin)	1. Ultra-small NLC improved CoQ10 skin permeation 2. Deeper penetration was obtained with ultra-small NLC	[148]
1. CoQ10 2.4 and 4.8% (*w*/*w*) 2. *Lipid phase:* cetyl palmitate and Miglyol 812 3. *Aqueous phase:* Tego Care 450 and water	1. Entrapment efficiency 2. In vitro release (Franz diffusion cells, cellulose acetate membrane)	1. Entrapment efficiency was 100% 2. Fast release observed at the initial stage followed by a prolonged release	[149]
1. CoQ10 2–5% 2. Lipid phase: glycerin monostearate, glyceride, and ethanol 3. Aqueous phase: Span 20 and water	1. Centrifugal stability 2. Radical scavenging assay (hydroxyl radical, superoxide anions, and DPPH radical)	1. No stratification phenomena were observed 2. Scavenging activity was enhanced with increasing CoQ10 loading	[150]
1. CoQ10 2–5% 2. *Lipid phase:* glycerin monostearate, glyceride and ethanol 3. *Aqueous phase:* Span 20 and water	1. Comparison between CoQ10-NLC and CoQ10 cosmetic on radical scavenging (hydroxyl radical, superoxide anions, and DPPH radical)	1. CoQ10-NLC exhibited higher antioxidant activity than CoQ10 cosmetic	[151]
1. CoQ10 2–5% 2. *Lipid phase:* glycerin monostearate, octyl and decyl glycerate, glyceride, and ethanol 3. *Aqueous phase:* Span 20 and water	1. In vitro release (dialysis bag) 2. In vitro cell viability (MTT assay, HaCaT cells) 3. Cell morphology (HaCaT cells, hematoxylin-eosin staining) 4. Cell nucleus morphology (HaCaT cells, Hoechst33342 staining) 5. Cell behavior (time-lapse imaging, HaCaT cells)	1. Prolonged-release was observed 2. CoQ10-NLC exhibited good cytocompatibility 3. Cells exhibited a typical morphology confirming cell viability study 4. Changes in structure, size, and morphology of cell nucleus were not detected 5. Time-lapse imaging confirmed cell viability results	[152]
1. CoQ10 (not reported) 2. *Lipid phase:* glycerin monostearate, octyl, and decyl glycerate, glyceryl triacetate, and glyceride 3. *Aqueous phase:* Span 20 and water	1. In vitro skin penetration (Franz diffusion cells, rabbit skin) 2. Cell viability (MTT assay, human keratinocytes HaCaT cells) 3. Cell morphology (time-lapse imaging assay) 4. Levels of oxidative stress markers (reactive oxygen species (ROS), superoxide dismutase (SOD), glutathione peroxidase (GSH-PX), and malondialdehyde (MDA))	1. NLC increased CoQ10 deposition into skin 2. Cell viability and morphology assays showed protective activity of CoQ10-NLC against oxidative damage 3. CoQ10-NLC attenuated generation of ROS, reestablished SOD and GSH-PX activities, and diminished lipid peroxidation via MDA inhibition	[153]
1. CoQ10 5% (*w*/*w*) 2. *Lipid phase:* octyl decyl acid glyceride 3. *Aqueous phase:* soybean lecithin, glycerol, and water	1. UVA irradiation (human embryo skin fibroblasts) 2. Cell viability (MTT assay, human embryo skin fibroblasts) 3. Levels of oxidative stress markers (reactive oxygen species (ROS), malondialdehyde (MDA), superoxide dismutase (SOD), and glutathione peroxidase (GSH-PX)) 4. Cell morphology and apoptosis or necrosis 5. In vivo skin penetration (Sprague-Dawley, Nile Red)	1. CoQ10-NLC demonstrated a protective effect 2. Cell viability increased after treatment with CoQ10-NLC 3. ROS and MDA levels decreased, and SOD and GSH-PX activities increased 4. CoQ10-NLC decreased apoptosis and necrosis 5. Skin penetration was improved	[154]

**Table 7:** An overview of CoQ10 encapsulation in nanostructured lipid carriers (NLC) employing other preparation methods.

Ingredients and Method	Parameters	Outcomes	Reference
1. CoQ10 500 mg 2. *Lipid phase:* Precirol ATO 5 and Miglyol 812 3. *Aqueous phase:* Lutrol F68, Tween 80 and water 4. Ultrasonication	1. CoQ10 loading 2. Cell cytotoxicity (MTT assay, human dermal fibroblasts) 3. Intracellular reactive oxygen species (ROS) assay 4. Mitochondrial membrane potential assay	1. CoQ10 recovery was > 84% 2. Reduced CoQ10-NLC prevented the loss in cell viability 3. ROS formation was counteracted by reduced CoQ10-NLC 4. Reduced CoQ10-NLC counteracted UVA-associated mitochondrial depolarization	[15]
1. CoQ10 (4%) and retinaldehyde (0.05%) 2. Lipid phase: Compritol 888 ATO and isopropyl myristate 3. Aqueous phase: Poloxamer F68 and water 4. High-shear homogenization	1. Entrapment efficiency 2. Rheological studies 3. Stability study (3 months) 4. In vitro release (dialysis membrane) 5. Cellular uptake (human keratinocytes HaCaT cells) 6. Cell cytotoxicity (MTT assay, HaCaT cells) 7. Ex vivo skin permeation (Franz diffusion cells, pig ear skin) 8. Dermal pharmacokinetics (tape stripping, pig ear skin) 9. Skin distribution (confocal laser scanning microscopy) 10. In vivo skin irritation (female Sprague Dawley rats) 11. Therapeutic efficacy (female Swiss albino mice)	1. Entrapment efficiency was > 80% 2. Slight decrease in spreadability 3. No significant change was observed in CoQ10 content 4. Fast release observed at the initial stage followed by a prolonged release 5. Cell uptake was observed 6. NLC were well tolerated by skin cells 7. Negligible permeation was observed 8. NLC demonstrated appreciable penetration 9. NLC distribution into the skin layer was observed 10. CoQ10 and retinaldehyde loaded into NLC were less irritant 11. Significant decrease in wrinkles were observed	[18]
1. CoQ10 2.8% 2. *Lipid phase:* cetyl palmitate and Labrafac Lipophile WL1349 3. *Aqueous phase:* Tego Care 450 and water 4. High-pressure microfluidics	1. CoQ10 loading and entrapment efficiency 2. Long-term stability (1 year) 3. In vitro release (dialysis bag) 4. In vitro skin permeation (Franz diffusion cells, abdomen skin SD rats)	1. CoQ10 loading was 2.51% and entrapment efficiency 100% 2. Physical parameters slightly changed during storage, but CoQ10 loading decreased 40% 3. Fast release observed at the initial stage followed by a prolonged release 4. An increase in CoQ10 concentration in the epidermis was observed	[124]
1. CoQ10 2.4% 2. Lipid phase: cetyl palmitate and alpha tocopheryl acetate 3. Aqueous phase: propylene glycol, Tween 80, pH phosphate buffer and water 4. High-shear homogenization	1. Entrapment efficiency 2. In vivo skin penetration (Male Wistar rats)	1. Entrapment efficiency was >70% 2. Penetration depth was influenced by the composition of the lipid phase	[126]
1. CoQ10/*Myrica esculenta* extract (1:1), 10% 2. *Lipid phase:* Precirol ATO 5 and oleic acid 3. *Aqueous phase:* Poloxamer 407 and water 4. Solvent injection	1. Optimization of the NLC composition 2. Entrapment efficiency 3. Stability study (28 days)	1. High lipid content and low surfactant concentration resulted in small particles 2. Entrapment efficiency was > 80% 3. Formulations exhibited good physical stability	[155]
1. CoQ10/*Myrica esculenta* extract (1:1), 10% 2. *Lipid phase:* Precirol ATO 5 and oleic acid 3. *Aqueous phase:* Poloxamer 407 and water 4. Solvent injection	1. In vitro permeation study (Franz diffusion cells, skin of albino Wistar rat) 2. In vitro photoprotective activity (albino Wistar rats)	1. An enhance in skin permeation was observed 2. NLC gel exhibited photoprotective activity	[156]
1. CoQ10 5% 2. *Lipid phase:* solid lipid (Precirol ATO 5 or Compritol 888 ATO), Captex 500, and polyvinyl alcohol 3. *Aqueous phase:* water or Poloxamer 188, mannitol, and water 4. Solvent diffusion	1. Entrapment efficiency 2. Stability study (90 days) 3. In vitro release (USP apparatus) 4. Antioxidant activity (DPPH assay)	1. Entrapment efficiency was > 62% 2. Slightly change in particle size was observed 3. Fast release observed at the initial stage followed by a prolonged release 4. CoQ10-NLC showed antioxidant activity	[157]
1. CoQ10 1% 2. *Lipid phase:* cetyl palmitate and olive oil 3. *Aqueous phase (A):* Tween 80 and Span 80 4. *Aqueous phase (B):* ethanol and acetate buffer pH 4.2 5. High-shear homogenization	1. In vivo skin fibroblast and collagen density (*Mus musculus*) 2. In vivo skin irritability (*Mus musculus*)	1. NLC improved the number of fibroblast cells and collagen density 2. No irritability effect was observed	[158]

## 7. Challenges on the Encapsulation of Carotenoids and Coenzyme Q10 in Lipid Nanoparticles

The occlusive effect of lipid nanocarriers over the skin, especially for reduced particle sizes, is an important feature to enhance bioactive permeation through the stratum corneum [159]. However, for cosmetic applications, deep permeation could be a drawback since systemic effects are not allowed for this class of products. Size and permeation tests are necessary to support cosmetic claims. Regarding stability, most studies evaluate the SLN/NLC dispersions itself over a short period of time. However, colloidal systems are sensible with the surrounding compounds, especially for other lipophilic molecules. Preservatives showed a great impact over nanocarriers agglomeration. However, the presence of stabilizers acting as anchors in the nanocarrier’s surface may help prevent the impairment of stability [147]. The behavior of SLN/NLC in more complex matrices (such as emulsions) may represent a limitation for commercial applications. In addition, for carotene nanocarriers, the intense color could also represent a drawback in consumer’s opinion.

## 8. Future Perspectives

The association of antioxidants with complementary mechanisms is a frequent strategy in dermatology to widen the neutralizing effect on oxidative stress. A mixture of vitamin E, vitamin C, plant extracts, and carotenes applied topically showed good outcomes in extrinsic skin aging [160]. However, the mixture of several molecules with different physical-chemical characteristics entrapped inside SLN/NLC nanocarriers would represent a technical challenge. The association of CoQ10 and vitamin E in nanocapsules showed anti-edematogenic, anti-inflammatory, and antioxidant effects in animal models against UVB radiation [161]. Since both molecules are lipophilic, their use in SLN and NLC could be developed to enhance skin delivery. In fact, NLC co-loading of idebenone (an analog of CoQ10) and vitamin E protected fibroblast cells from oxidative stress, reduced skin pigmentation, and increased skin hydration [78].

Kinetics studies regarding drug release of SLN/NLC mimicking real usage could bring important data on the safety of those nanocarriers, especially to treat impaired skin. The faster release was observed in studies with impaired animal skin models [162]. The release profile is information to design nanocarrier for specific treatments, such as atopic dermatitis and other skin disorders that would beneficiate from the moisturizing effects of SLN/NLC. 

The use of green surfactants to reduce environmental impact could also be addressed in further investigations regarding SLN and NLC development, along with the reduction of solvent usage and high energy methods. The biosurfactant sophorolipid showed promising results for lipid nanocarriers [163]. Surfactants are also used to functionalize the nanocarrier’s surface, along with small molecules, polymers, and biomolecules, depending on the application [164]. For topical use, there is an opportunity to investigate the impact of functionalization since there is scarce literature addressing this issue.

## 9. Concluding Remarks

Topical administration of carotenoids and CoQ10 can offer several benefits, although the skin barrier makes it difficult for these molecules to penetrate and permeate through the skin. From the studies described in this review, it was demonstrated that loading carotenoids and CoQ10 into lipid nanoparticles is an attractive strategy to increase the topical effectiveness of these bioactive compounds by improving their bioavailability, chemical stability, and skin absorption. Overall, an increase in these parameters was observed. Incorporation into lipid nanoparticles protected carotenoids and CoQ10 from degradation. It is important to note that carotenoid stability was affected by NLC oil content and process temperature. CoQ10 was stable in the short-term (3 months) while long-term (1 year) studies resulted in different outcomes. Studies on skin absorption were mainly performed in animal skin models, whereas few experiments were conducted in human skin. The studies demonstrated that carotenoids and CoQ10 penetrated the skin and were retained in the epidermis and dermis. Additionally, it was shown that the lipid matrix (composition and oil content) affected skin absorption.

Regarding the antioxidant and photoprotective properties, most systems demonstrated high antioxidant capacity under several oxidative conditions. Similarly, the majority of the systems showed good photoprotective activity. Other important findings were the low cytotoxic effect, high biocompatibility, and good anti-inflammatory activity demonstrated by the systems. Owing to these results, topical administration of carotenoids and coenzyme Q10 encapsulated into lipid nanoparticles could be considered an appealing strategy to protect skin from photodamages.

## Figures and Tables

**Table 1:** Studies on carotenoid encapsulation in solid lipid nanoparticles (SLN) employing high-pressure homogenization as the preparation method.

Formulation Ingredients	Parameters	Outcomes	Reference
1. Beta-carotene 0.1% (*w*/*w*) 2. *Lipid phase:* canola stearin 3. *Aqueous phase:* Poloxamer 188 or Tween 20 and water	1. Surfactant effect 2. Beta-carotene stability	1. Polymorphism was affected by surfactant type 2. Beta-carotene degradation was minimized	[10]
1. Lutein 1% (*w*/*w*) 2. *Lipid phase:* cetyl palmitate, glyceryl tripalmitate or carnauba wax 3. *Aqueous phase:* Plantacare 810 and water	1. Chemical and short-term (1 month) stability 2. In vitro release (Franz diffusion cells, membrane-free model) 3. In vitro penetration study (Franz diffusion cells, synthetic cellulose nitrate membrane, or fresh dermis of pig ear skin) 4. Photostability studies using a solar simulator	1. Lutein prepared with carnauba wax showed the highest thermostability. Significant changes were observed in particle size at high temperature 2. It was observed lower release 3. SLN remained in the upper layers of the skin (pig ear skin) 4. SLN showed good photostability	[9] ^a^
1. Lycopene 5 mg 2. *Lipid phase:* orange wax 3. *Aqueous phase:* Eumulgin SG and water	1. Comparison of the internal structure of SLN and NLC nanocarriers	1. Addition of rice bran oil-modified the crystalline characteristics of orange wax	[11] ^a^
1. Lycopene 0.005% (*w*/*w*) 2. *Lipid phase:* orange wax 3. *Aqueous phase:* Eumulgin SG and water	1. Comparison of the internal structure of SLN and NLC nanocarriers	1. Addition of cholesterol to the lipid phase decreased particle sizes	[111] ^a^

^a^ The studies also evaluated NLC system.

**Table 2:** Studies on carotenoid encapsulation in solid lipid nanoparticles (SLN) employing other preparation methods.

Ingredients and Method	Parameters	Outcomes	Reference
1. Beta-carotene 0.055 and 0.086 g 2. *Lipid phase:* stearyl ferulate or stearic acid 3. *Aqueous phase:* sodium taurocholate, butanol, Tween 20, and water 4. Microemulsion	1. Antioxidant properties (rat-liver microsomal membranes) 2. Entrapment efficiency 3. Stability assay (3 months) 4. Cytotoxic effect (MTT assay, RAT-1 cells)	1. Both SLN showed high antioxidant properties 2. Entrapment efficiency was > 48% for stearyl ferulate-based SLN 3. A slight increase in particle size was observed 4. High doses of SLN exerted a cytotoxic effect	[112]
1. Beta-carotene 1 mg/g total lipid 2. *Lipid phase:* eicosane 3. *Aqueous phase:* lecithin, bile salts, and water 4. High shear homogenization and ultrasound	1. Loading efficiency 2. Beta-carotene stability (radical mediated, AAPH)	1. Beta-carotene entrapment increased as oil content increased 2. Beta-carotene stability improved as oil content increased	[113] ^a^
1. Crocin 0.1 g and crocetin 0.064 g obtained from the hydrolysis of crocin 2. *Lipid phase (crocin):* Softisan 100, ethanol, and water 3. *Aqueous phase (crocin):* Pluronic F68 and water 4. *Lipid phase (crocetin):* Softisan 100 and ethanol 5. *Aqueous phase (crocetin):* hydroxypropyl methyl cellulose, Pluronic F68, soy lecithin, and water 6. Quasi-emulsion solvent diffusion and solvent diffusion method	1. Entrapment efficiency 2. Stability assay (Turbiscan Lab) 3. In vitro release (glass vials) 4. Oxygen radical absorbance assay 5. In vitro cytotoxic effect (Human melanoma A375 and malignant Schwann sNF96.2 cell lines, MTT assay)	1. Entrapment efficiency was > 80% 2. Incorporation of active ingredient increased SLN physical stability 3. Fast release observed at the initial stage followed by a prolonged release 4. Crocetin showed higher antioxidant activity than crocin 5. SLN-based formulations showed different antiproliferative effect against the A375 and sNF96.2 cell lines	[12]

^a^ Comparative study between SLN and NLC systems.

**Table 3:** Studies on carotenoid encapsulation in nanostructured lipid carriers (NLC) employing high-pressure homogenization as the preparation method.

Formulation Ingredients	Parameters	Outcomes	Reference
1. Carotenoid mixture 147–350 mg carotenoids/100 g oil (Carrot extract) 2. *Lipid phase:* glycerol monostearate, cetyl alcohol, and beeswax 3. *Aqueous phase:* Tween 20, phosphatidylcholine and Synperonic PE/F68	1. Entrapment efficiency 2. In vitro release (Franz diffusion cells, Tuffryn membrane) 3. In vitro antioxidant activity (chemiluminescence) 4. In vitro cell viability (mouse fibroblast cell line NCTC clone L929, MTT assay)	1. Entrapment efficiency was > 78% 2. Fast release at the initial stage followed by a prolonged release 3. High antioxidant activity was observed 4. NLC demonstrated low cytotoxicity	[114]
1. Carotenoid mixture 0.037–0.11% (*w/w*), Marigold extract 2. *Lipid phase:* mixture of solid lipids (glycerol monostearate and cetyl palmitate) and vegetable oils (amaranth and hempseed) 3. *Aqueous phase:* Tween 20, phosphatidylcholine, Synperonic PE/F68, and water	1. Stability and entrapment efficiency 2. In vitro antioxidant activity (chemiluminescence) 3. In vitro release (Franz diffusion cells, cellulose nitrate membrane)	1. NLC stability was unaffected by carotenoid incorporation 2. NLC containing mixtures of the vegetable oils showed better entrapment efficiency 3. Carotenoid-NLC demonstrated high antioxidant activity 4. Gradual release was observed for NLC containing vegetable oil mixtures	[115] ^a^
1. Carotenoid mixture 0.86% (*w*/*w*), Marigold extract with 210 mg carotenoids/100 g oily fraction, and azelaic acid 1% 2. *Lipid phase:* glycerol monostearate and cetyl palmitate 3. *Aqueous phase:* Tween 20, phosphatidylcholine, Poloxamer 188, and water	1. Entrapment efficiency 2. In vitro cell viability (fibroblast L929 cell line, MTT assay) 3. In vitro anti-inflammatory activity (human monocytic leukemia cell line THP-1) 4. In vivo anti-inflammatory action (Male Wistar rats)	1. Entrapment efficiency was > 90% 2. High biocompatibility was observed 3. Reduced expression of inflammatory cytokines 4. Edema was significantly reduced	[116]
1. Carotenoid mixture 6%, Marigold and carrot extracts with 210 mg carotenoids/100 g oily fraction and azelaic acid 2% 2. *Lipid phase:* glycerol monostearate and cetyl palmitate 3. *Aqueous phase:* Tween 20, phosphatidylcholine, Poloxamer 188, and water	1. In vitro and In vivo assessment of the topical formulations	1. Topical formulations demonstrated high biocompatibility, significant antimicrobial and antioxidant activities, and improved anti-inflammatory and antiacne actions	[117]
1. Lutein 1% (*w*/*w*) 2. *Lipid phase:* glyceryl tripalmitate/Miglyol 812 or carnauba wax/Miglyol 812 3. *Aqueous phase:* Plantacare 810 and water	1. Chemical and short-term (1 month) stability 2. In vitro release (Franz diffusion cells, membrane-free model) 3. In vitro penetration study (Franz diffusion cells, synthetic cellulose nitrate membrane or fresh dermis of pig ear skin) 4. Photostability studies using solar simulator	1. Lutein prepared with carnauba wax showed the highest thermostability. Significant changes were observed on particle size at high temperature 2. Higher penetration was obtained for NLC (membrane) 3. NLC remained in the upper layers of the skin (pig ear skin) 4. NLC showed good photostability	[9] ^b^
1. Lycopene 5 mg 2. *Lipid phase:* orange wax and rice bran oil 3. *Aqueous phase:* Eumulgin SG and water	1. Comparison of the internal structure of SLN and NLC nanocarriers	1. Addition of rice bran oil modified the crystalline characteristics of orange wax	[11] ^b^
1. Lycopene 0.005% (*w*/*w*) 2. *Lipid phase:* orange wax/rice oil and orange wax/rice oil/cholesterol 3. *Aqueous phase:* Eumulgin SG and water	1. Effect of the lipid mixture 2. Stability assay (45 days)	1. Addition of cholesterol to the lipid phase decreased particle sizes 2. NLC without cholesterol and storage below room temperature protected lycopene from degradation	[111] ^b^
1. Lycopene 5, 25 and 50 mg 2. Lipid phase: orange wax and rice bran oil 3. Aqueous phase: Eumulgin SG and water	1. Entrapment efficiency 2. In vitro release (glass tube) 3. Antioxidant activity (ABTS and DPPH methods) 4. Stability assay (4 months)	1. Entrapment efficiency was close to 100% 2. Fast release observed at the initial stage followed by a prolonged release 3. NLC formulations showed free radical scavenging activities 4. NLC minimized lycopene degradation	[118]
1. Lycopene 10% (*w*/*w*) 2. *Lipid phase:* orange wax and rosemary oil 3. *Aqueous phase:* surfactant (Plantacare 1200, C-1216, C-1816, C-1616 or C-1815) and water	1. Contact angle measurement (goniometry) 2. Particle size 3. Stability assay (1 month) 4. Chemical stability	1. The surfactants exhibited different spreading and wetting properties 2. Particle size was influenced by the surfactant type 3. Plantacare 1200 was the most suitable surfactant for NLC 4. Lycopene stability was significantly enhanced	[119]

^a^ The study also employed high shear homogenization as a preparation method. ^b^ The studies also evaluated the SLN system.

**Table 4:** Studies on carotenoid encapsulation in nanostructured lipid carriers (NLC) employing other preparation methods.

Ingredients and Method	Parameters	Outcomes	Reference
1. Astaxanthin 103 mg 2. *Lipid phase:* Precirol ATO 5 and Tween 80 3. *Aqueous phase:* Poloxamer 407 and water 4. Hot homogenization	1. Astaxanthin content 2. Antioxidant activity (alpha-tocopherol equivalent antioxidant capacity)	1. Astaxanthin content remained at 90% after 30 days 2. Astaxanthin antioxidant activity was enhanced	[13]
1. Beta-carotene 1 mg/g total lipid 2. *Lipid phase:* glyceryl trioctanoate and eicosane 3. *Aqueous phase:* lecithin, bile salts, and water 4. High shear homogenization and ultrasound	1. Loading efficiency 2. Beta-carotene stability (radical mediated, AAPH)	1. Beta-carotene entrapment increased as oil content increased 2. Beta-carotene stability improved as oil content increased	[113] ^a^
1. Beta-carotene 34.56, 51.13 and 53.97 ppm 2. *Lipid phase:* hydrogenated palm kernel glycerides, isopropyl palmitate, and Span 40 3. Aqueous phase: Tween 80 and water 4. Hot homogenization	1. Modification of the heating temperature of the lipid and aqueous phases 2. Beta-carotene content 3. Accelerated stability test (3 months)	1. High production temperature lead to an increase in particle size 2. Low beta-carotene content was determined when high temperature was employed 3. Phase separation was observed for NLC prepared at high temperature after 2-month storage at 45 °C. No crystallization or gel formation was observed after 3-month storage at 5 °C	[120]
1. Carotenoid mixture 0.037–0.11% (*w/w*), Marigold extract 2. *Lipid phase:* mixture of solid lipids (glycerol monostearate and cetyl palmitate) and vegetable oils (amaranth and hempseed) 3. *Aqueous phase:* Tween 20, phosphatidylcholine, Synperonic PE/F68, and water 4. High shear homogenization	1. Stability and entrapment efficiency 2. In vitro antioxidant activity (chemiluminescence) 3. In vitro release (Franz diffusion cells, cellulose nitrate membrane)	1. NLC stability was unaffected by carotenoid incorporation 2. NLC containing mixtures of the vegetable oils showed better entrapment efficiency 3. Carotenoid-NLC demonstrated high antioxidant activity 4. Gradual release was observed for NLC containing vegetable oil mixtures	[115] ^b^
1. Fucoxanthin 0.05% (*w*/*w*) 2. *Lipid phase:* bacuri butter, tucumã oil, and sorbitan monooleate 3. *Aqueous phase:* polysorbate 80 and water 4. High shear homogenization	1. Entrapment efficiency 2. Bioadhesion properties 3. In vitro cellular uptake (fibroblasts) 4. Pharmacological activity in the psoriatic-like cellular model (human keratinocyte, HaCaT)	1. Entrapment efficiency close to 100% 2. NLC coated with chitosan showed higher adhesion 3. Cellular uptake was observed 4. Fucoxanthin loaded in NLC reduced skin hyperproliferation and inflammation	[121]
1. Fucoxanthin 0.05% (*w*/*w*) 2. *Lipid phase:* bacuri butter, tucumã oil, and sorbitan monooleate 3. *Aqueous phase:* polysorbate 80 and water 4. High shear homogenization	1. Photostability assay 2. Accelerated stability (45 days) 3. In vitro dissolution (glass tube) 4. In vitro skin permeation (Franz diffusion cells, porcine ears)	1. NLC protected fucoxanthin against degradation 2. Stability was observed in all temperatures 3. NLC promoted a controlled release 4. Fucoxanthin was retained in the epidermis and dermis	[122]
1. Lutein 6, 10, and 15 mg 2. Lipid phase: palmitic acid, Lipoid S100, and ethanol 3. Aqueous phase: water 4. Solvent injection method	1. Stability assay (Turbiscan Lab) 2. In vivo anti-inflammatory activity (human volunteers) 3. In vivo tolerability (human volunteers) 4. In vitro percutaneous absorption (Franz diffusion cells, human stratum corneum, and viable epidermis)	1. NLC containing a great amount of lutein showed high stability over time 2. NLC containing a low amount of lutein protected the skin against induced erythema 3. All formulations were well tolerated 4. Similar permeation profile was observed	[123]

^a^ The studies also evaluated SLN. ^b^ The study also employed high-pressure homogenization as a preparation method.
